# Association between constipation and major depression in adult Americans: evidence from NHANES 2005–2010

**DOI:** 10.3389/fpsyt.2023.1152435

**Published:** 2023-08-15

**Authors:** Pengfei Wang, Xia Shen, Yan Wang, Xiaoqiang Jia

**Affiliations:** ^1^Department of Anorectal Surgery, China Academy of Chinese Medical Sciences Xiyuan Hospital, Beijing, China; ^2^Department of Nursing, Wuxi Medical College, Jiangnan University, Wuxi, China; ^3^Department of Oncology, China Academy of Chinese Medical Sciences Guang’anmen Hospital, Beijing, China

**Keywords:** constipation, major depression, female, cross-sectional study, NHANES

## Abstract

**Objective:**

Current studies on the association between constipation and depression is still insufficient. In this study, we investigated the detailed association between constipation and major depression among American adults.

**Methods:**

In this cross-sectional study, 12,352 adults aged 20 and older were selected from the National Health and Nutrition Examination Survey 2005–2010 for the sample. Constipation was defined as fewer than three defecation frequencies per week. For the assessment of major depression, the validated Patient Health Questionnaire-9 was used. Adjusted odds ratios (ORs) were calculated using multivariate logistic regression models. A subgroup analysis was carried out to ensure that the results were stable.

**Results:**

Of the 12,352 participants, 430 reported constipation, with a prevalence of 3.5%. Depression was reported in 1030 cases, indicating a prevalence rate of 8.3%. Patients with constipation were significantly more likely to have major depression (20.9%) than those without it (7.9%, *p* < 0.001). After adjusting for age, sex, race/ethnicity, marital status, education level, body mass index, vigorous physical activity, alcohol consumption, smoking status, poverty income ratio, diabetes, selective serotonin reuptake inhibitor use, liver disease, heart disease, pulmonary disease, hypertension, arthritis, cancer, dietary fiber intake, moisture intake, total fat intake, carbohydrates intake, and protein intake, constipation is significantly associated with major depression (OR: 2.20, 95%CI: 1.68–2.87, *p* < 0.001). Subgroup analyses by age, sex, dietary intake, risk behaviors, and common complications showed no statistically significant interactions (*p* > 0.05).

**Conclusion:**

In conclusion, this study showed that constipation were significantly associated with depression. When treating patients with constipation, it is necessary for clinicians to screen and evaluate depression, and provide timely and effective intervention for patients with depression to avoid further deterioration of the condition.

## 1. Introduction

Depression, sometimes called major depressive disorder (MDD), is among the most commonly encountered psychiatric disorder, with reported 12 months and lifetime prevalence rates of 7.2 and 10.8% (1). It is one of the leading causes of disease burden or death worldwide (2). In terms of years lived with disability (YLDs) and disability-adjusted life-years (DALYs), depression ranks second and 13th among the leading causes, respectively (3), and the World Health Organization predicts that it will become the first disease burden in the world by 2030 (4). A meta-analysis of data from 35 countries found that people with depression had a 52% increased risk of death (5). Therefore, screening and early intervention for depression are very important, and one of the keys to screening and intervention is to determine the risk factors associated with depression and identify high-risk groups.

Constipation is characterized by low frequency of defecation, difficulty in defecation, and incomplete defecation, which seriously affects the quality of life (6). Limited studies reported that constipation was associated with depression, but the study populations were adolescents or older adults mostly. For example, one cross-sectional survey conducted in Sri Lanka found that adolescents (13 to 18 years old) with constipation were more likely to have psychological problems such as depression and anxiety (7). More recently, in a survey of Korean high school students (16 to 18 years old), constipation was also found to be independently associated with mild depression (8). A another study based on people aged 50 years and older in the United States showed that depression was a risk factor for constipation (9). Whether the results based on these specific age groups can be generalized to adults aged 20 years and older in the United States needs to be explored further.

Among Iranian adults aged 18–55, a study evaluated the factors associated with chronic constipation and found depression to be significantly influential (10). The study was based on an Asian Iranian population and did not involve participants from the American population. We cannot exclude the possibility that the association may differ by race/ethnicity. Additionally, the study did not encompass individuals above the age of 55. Another study, also utilizing data from NHANES, examined this subject among aged 20 years or older (11). In that study, however, constipation was only defined based on stool consistency using the Bristol Stool Form Scale, without accounting for stool frequency. The frequency of bowel movements represents a significant characteristic of constipation (6) and serves as a validated method for defining constipation in the NHANES dataset (12, 13). Studies shown that stool consistency and stool frequency correlated very weakly with one another (14). Thus, further investigation into this association is necessary by considering stool frequency as a defining criterion for constipation.

Constipation is highly prevalent in the adult general population: the estimated prevalence can be as high as 2 to 27% (15). Studies shown that depression can reduce patients’ adherence to constipation treatment (16), and the use of some antidepressant drugs may also aggravate constipation symptoms (17). Therefore, it is necessary to pay attention to the mental health of constipated patients. Further research on the association between constipation and depression will help to understand the psychological characteristics of patients with constipation and provide more comprehensive information for clinicians to treat constipated patients with depression. Therefore, study objectives were to further examine whether there was an significant association between constipation and major depression in US adults.

## 2. Materials and methods

### 2.1. Data source

The National Health and Nutrition Examination Survey (NHANES), conducted by the National Center for Health Statistics (NCHS) of the Centers for Disease Control and Prevention (CDC), is a cross-sectional survey of the uninstitutionalized civilian population in the United States (18). Physical examinations, interviews, and laboratory assessments are conducted at mobile examination centers (MECs), after demographic, socioeconomic, and medical health interviews are conducted at homes. If the topics discussed were sensitive, participants were interviewed privately in the MEC. With informed consent from all participants, the protocol was approved by the NCHS Ethics Review Board.

**Figure 1:**
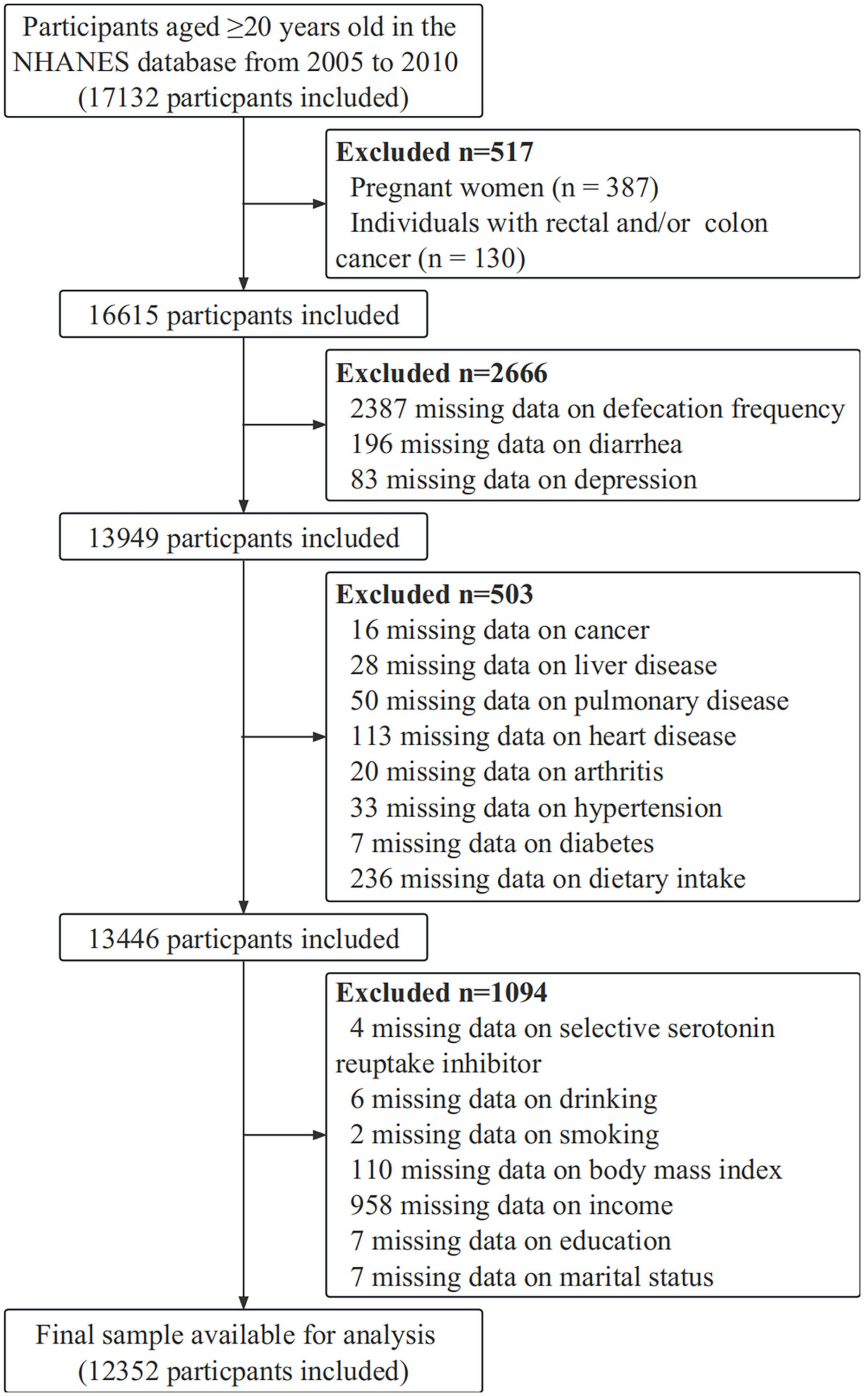
Flow diagram of the sample selection from the National Health and Nutrition Examination Survey (NHANES) 2005–2010.

Because constipation data were only available in the NHANES 2005–2010, the data for this study were extracted only from adult participants aged ≥20 years in these three 2 year cycles. Individuals were excluded if they had rectal and/or colon cancer or if they were pregnant (19–21). Ultimately, 12,352 participants were included in the analyses. As shown in Figure 1, the patient selection process is illustrated by a flow diagram.

### 2.2. Constipation

Defecation frequency and stool consistency were used by NHANES to measure constipation in participants who answered the bowel health questionnaire. Based on NHANES data, defecation frequency was used to define constipation since stool frequency and consistency were poorly correlated (14). During the survey, participants were asked to estimate how often they had bowel movements each week. Among the responses, less than 3 bowel movements a week were classified as constipated, 3 to 21 bowel movements a week were classified as normal, and more than 21 bowel movements a week were classified as diarrhea in line with previous NHANES data (12, 13).

### 2.3. Major depression

Using PHQ-9 (Patient Health Questionnaire-9), major depression was identified in MECs during private interview sessions. In the PHQ-9, nine items are scored on a four-point scale (0 = never; 3 = almost every day), with scores ranging from 0 to 27. Our study defined major depression with scores ≥10 according to the previous paper (9, 22–24).

### 2.4. Covariates

Several covariables were evaluated as possible factors associated with depression and constipation (11). The covariates in this study included age (continuous variable), education level, race/ethnicity, sex, body mass index (BMI), marital status, vigorous physical activity, alcohol intake, smoking status, poverty income ratio (PIR), selective serotonin reuptake inhibitor (SSRI) use, reported medical comorbidities, and dietary intake. Education level was grouped as “≤ high school” and “> high school.” BMI was divided into three groups: under/normal weight (< 25.0 kg kg/m^2^), overweight (25.0–29.9 kg/m^2^), and obese (≥ 30 kg/m^2^) (11, 25, 26). Ethnicity and race were categorized into four groups: Hispanic, White, Black, and other. The PIR was divided into <2 and ≥ 2 times the poverty threshold. There were three categories of marital status: married or living together; divorced, separated, or widowed; and never married. Vigorous physical activity was classified as vigorous-intensity activity that causes large increases in breathing or heart rate like carrying or lifting heavy loads, digging, or construction work for at least 10 min continuously. Among participants who replied “Do you smoke now?” three groupings were formed: current, former, and never smokers. “Never smokers” are interviewees who have never smoked or smoked fewer than 100 cigarettes before the interview. “Former smokers” were defined as those who smoked more than 100 cigarettes at the time of their interview. The term “current smoker” refers to someone who has smoked at least 100 cigarettes in their lifetime while still smoking at the time of the interview. A drinker is someone who consumes at least 12 drinks annually. SSRI use was classified as “yes” or “no” based on participant report. During the 24 h dietary recall conducted by the NHANES from 2005–2010, dietary fiber, moisture, total fat, carbohydrate, and protein intake were collected. In order to determine the distribution of these dietary variables, quartiles were used. According to the Medical Conditions Questionnaire, medical comorbidities were also included. Among the associated comorbid conditions were diabetes, liver disease, heart disease (angina, chronic heart failure, coronary disease or myocardial infarction), pulmonary disease (emphysema, asthma or chronic bronchitis), hypertension, arthritis and cancer.

### 2.5. Statistical analyses

An analysis of descriptive nature was performed on all participants. Continuous data from all participants were analyzed using mean, standard deviation (SD), or median, interquartile range (IQR) depending on the type of data. Categorical variables were represented by a percentage (%). An analysis of categorical variables was conducted using the Chi-square test. Continuous variables (age) were analyzed by *T*-test. Models of logistic regression were used to examine the relationship between constipation and major depression. Both non-adjusted and multivariate adjusted models were used: Model I, without adjustment for any covariates; Model II, sex and age were adjusted; Model III adjusted for covariates in Model II and race/ethnicity, marital status, education level, PIR, BMI, vigorous physical activity, selective serotonin reuptake inhibitor (SSRI) use, smoking status, and alcohol consumption; Model IV adjusted for covariates in Model III and diabetes, hypertension, arthritis, heart disease, pulmonary disease, liver disease, and cancer. Model V adjusted for covariates in Model IV and dietary fiber, moisture, total fat, carbohydrate, and protein intake. Subgroup and interaction analyses allied to age, sex, dietary intake, risk behaviors, SSRI use, and common complications were also performed to test the stability of the association between constipation and depression. Statistical significance was determined by comparing adjusted odds ratios (ORs) with 1.0 and describing 95% confidence intervals (CIs).

The statistical software packages R (http://www.R-project.org, The R Foundation) and Free Statistics software versions 1.7 were used for all the analyses. A two-tailed test was conducted and *p* < 0.05 was considered statistically significant.

## 3. Results

### 3.1. Baseline characteristics

In Table 1, we compared the characteristics of participants in the constipated and normal groups. Among these participants, 430 reported constipation, with a prevalence of 3.5%. Patients with constipation had a higher incidence of being younger, female, White, divorced, separated or widowed, never married, under/normal weight, smokers, with a lower education level, and PIR. Constipated individuals consumed less fiber, fat, protein, carbohydrates, and water in their diets. Constipation was associated with a greater likelihood of heart disease and pulmonary disease among patients with comorbidities. 1,030 reported depression, with a prevalence of 8.3%. Patients with constipation were significantly more likely to have major depression (20.9%) than those without it (7.9%, *p* < 0.001).

**Table 1:** Characteristics of participants in the constipated and normal groups.

Variables	Total	Normal	Constipated	*p*
	*n* = 12,352	*n* = 11,922	*n* = 430	
Age (years, mean ± SD)	49.3 ± 17.7	49.4 ± 17.7	44.9 ± 17.5	<0.001
Sex, *n* (%)				<0.001
Male	6,215 (50.3)	6,117 (51.3)	98 (22.8)	
Female	6,137 (49.7)	5,805 (48.7)	332 (77.2)	
Race/ Ethnicity, *n* (%)				<0.001
White	6,248 (50.6)	6,050 (50.7)	198 (46.0)	
Black	2,498 (20.2)	2,347 (19.7)	151 (35.1)	
Hispanic	3,129 (25.3)	3,061 (25.7)	68 (15.8)	
Other	477 (3.9)	464 (3.9)	13 (3.0)	
Marital Status, *n* (%)				<0.001
Married or living with partner	7,568 (61.3)	7,345 (61.6)	223 (51.9)	
Divorced, separated, or widowed	2,764 (22.4)	2,651 (22.2)	113 (26.3)	
Never married	2020 (16.4)	1926 (16.2)	94 (21.9)	
Education Level, *n* (%)				0.001
≤ high school	6,276 (50.8)	6,025 (50.5)	251 (58.4)	
> high school	6,076 (49.2)	5,897 (49.5)	179 (41.6)	
Family PIR, *n* (%)				<0.001
< 2	5,592 (45.3)	5,329 (44.7)	263 (61.2)	
≥ 2	6,760 (54.7)	6,593 (55.3)	167 (38.8)	
BMI, *n* (%)				0.008
Under/normal weight (< 25.0)	3,573 (28.9)	3,420 (28.7)	153 (35.6)	
Overweight (25.0–29.9)	4,216 (34.1)	4,082 (34.2)	134 (31.2)	
Obese (≥30.0)	4,563 (36.9)	4,420 (37.1)	143 (33.3)	
Vigorous physical activity, *n* (%)				0.061
No	9,446 (76.5)	9,101 (76.3)	345 (80.2)	
Yes	2,906 (23.5)	2,821 (23.7)	85 (19.8)	
Smoking status, *n* (%)				<0.001
Never	6,443 (52.2)	6,213 (52.1)	230 (53.5)	
Former	3,134 (25.4)	3,058 (25.7)	76 (17.7)	
Current	2,775 (22.5)	2,651 (22.2)	124 (28.8)	
Alcohol intake, *n* (%)				<0.001
No	3,419 (27.7)	3,253 (27.3)	166 (38.6)	
Yes	8,933 (72.3)	8,669 (72.7)	264 (61.4)	
Diabetes, *n* (%)				0.313
No	10,986 (88.9)	10,610 (89.0)	376 (87.4)	
Yes	1,366 (11.1)	1,312 (11.0)	54 (12.6)	
Hypertension, *n* (%)				0.771
No	8,828 (71.5)	8,518 (71.4)	310 (72.1)	
Yes	3,524 (28.5)	3,404 (28.6)	120 (27.9)	
Arthritis, *n* (%)				0.359
No	8,982 (72.7)	8,661 (72.6)	321 (74.7)	
Yes	3,370 (27.3)	3,261 (27.4)	109 (25.3)	
Heart disease, *n* (%)				0.008
No	11,367 (92.0)	10,986 (92.1)	381 (88.6)	
Yes	985 (8.0)	936 (7.9)	49 (11.4)	
Pulmonary disease, *n* (%)				<0.001
No	10,199 (82.6)	9,872 (82.8)	327 (76.0)	
Yes	2,153 (17.4)	2050 (17.2)	103 (24.0)	
Liver disease, *n* (%)				0.963
No	11,945 (96.7)	11,529 (96.7)	416 (96.7)	
Yes	407 (3.3)	393 (3.3)	14 (3.3)	
Cancer, *n* (%)				0.853
No	11,234 (90.9)	10,844 (91.0)	390 (90.7)	
Yes	1,118 (9.1)	1,078 (9.0)	40 (9.3)	
Moisture intake, *n* (%)				<0.001
Lowest quartile	3,088 (25.0)	2,907 (24.4)	181 (42.1)	
Middle lower quartile	3,088 (25.0)	2,993 (25.1)	95 (22.1)	
Middle upper quartile	3,088 (25.0)	2,996 (25.1)	92 (21.4)	
Highest quartile	3,088 (25.0)	3,026 (25.4)	62 (14.4)	
Total fat intake, *n* (%)				<0.001
Lowest quartile	3,088 (25.0)	2,966 (24.9)	122 (28.4)	
Middle lower quartile	3,088 (25.0)	2,968 (24.9)	120 (27.9)	
Middle upper quartile	3,088 (25.0)	2,972 (24.9)	116 (27.0)	
Highest quartile	3,088 (25.0)	3,016 (25.3)	72 (16.7)	
Protein intake, *n* (%)				<0.001
Lowest quartile	3,085 (25.0)	2,922 (24.5)	163 (37.9)	
Middle lower quartile	3,091 (25.0)	2,970 (24.9)	121 (28.1)	
Middle upper quartile	3,088 (25.0)	3,004 (25.2)	84 (19.5)	
Highest quartile	3,088 (25.0)	3,026 (25.4)	62 (14.4)	
Dietary fiber intake, *n* (%)				<0.001
Lowest quartile	3,071 (24.9)	2,899 (24.3)	172 (40.0)	
Middle lower quartile	3,083 (25.0)	2,965 (24.9)	118 (27.4)	
Middle upper quartile	3,102 (25.1)	3,014 (25.3)	88 (20.5)	
Highest quartile	3,096 (25.1)	3,044 (25.5)	52 (12.1)	
Carbohydrate intake, *n* (%)				0.004
Lowest quartile	3,088 (25.0)	2,961 (24.8)	127 (29.5)	
Middle lower quartile	3,088 (25.0)	2,965 (24.9)	123 (28.6)	
Middle upper quartile	3,088 (25.0)	2,989 (25.1)	99 (23.0)	
Highest quartile	3,088 (25.0)	3,007 (25.2)	81 (18.8)	
SSRI use, *n* (%)				0.538
No	11,575 (93.7)	11,169 (93.7)	406 (94.4)	
Yes	777 (6.3)	753 (6.3)	24 (5.6)	
Major Depression, *n* (%)				<0.001
No	11,322 (91.7)	10,982 (92.1)	340 (79.1)	
Yes	1,030 (8.3)	940 (7.9)	90 (20.9)	

PIR, poverty income ratio; BMI, body mass index; SSRI, selective serotonin reuptake inhibitor use.

In Table 2, we compared the characteristics of the participants in the depressed and non-depressed groups. Patients with major depression had a higher incidence of being younger, female, black, hispanic, divorced, separated or widowed, never married, obese, smokers, SSRI use, with a lower education level, PIR, and percentage of vigorous physical activity practicing. Patients with major depression consumed less fiber, fat, protein, carbohydrates, and water in their diets. Among patients with comorbidities, major depression was more likely to be associated with diabetes, liver disease, heart disease, pulmonary disease, hypertension, and arthritis. Patients with major depression were significantly more likely to have constipation (8.7%) than those without it (3.0%, *p* < 0.001).

**Table 2:** Characteristics of the participants in the depressed and non-depressed groups.

Variables	Total	Non-depressed	Depressed	*p*
	*n* = 12,352	*n* = 11,322	*n* = 1,030	
Age (years, mean ± SD)	49.3 ± 17.7	49.5 ± 17.9	47.2 ± 15.4	<0.001
Sex, *n* (%)				<0.001
Male	6,215 (50.3)	5,839 (51.6)	376 (36.5)	
Female	6,137 (49.7)	5,483 (48.4)	654 (63.5)	
Race/ Ethnicity, *n* (%)				0.005
White	6,248 (50.6)	5,778 (51.0)	470 (45.6)	
Black	2,498 (20.2)	2,268 (20.0)	230 (22.3)	
Hispanic	3,129 (25.3)	2,835 (25.0)	294 (28.5)	
Other	477 (3.9)	441 (3.9)	36 (3.5)	
Marital Status, *n* (%)				<0.001
Married or living with partner	7,568 (61.3)	7,077 (62.5)	491 (47.7)	
Divorced, separated, or widowed	2,764 (22.4)	2,423 (21.4)	341 (33.1)	
Never married	2020 (16.4)	1822 (16.1)	198 (19.2)	
Education Level, *n* (%)				<0.001
≤ high school	6,276 (50.8)	5,619 (49.6)	657 (63.8)	
> high school	6,076 (49.2)	5,703 (50.4)	373 (36.2)	
Family PIR, *n* (%)				<0.001
< 2	5,592 (45.3)	4,871 (43.0)	721 (70.0)	
≥ 2	6,760 (54.7)	6,451 (57.0)	309 (30.0)	
BMI, *n* (%)				<0.001
Under/normal weight (< 25.0)	3,573 (28.9)	3,304 (29.2)	269 (26.1)	
Overweight (25.0–29.9)	4,216 (34.1)	3,930 (34.7)	286 (27.8)	
Obese (≥30.0)	4,563 (36.9)	4,088 (36.1)	475 (46.1)	
Vigorous physical activity, *n* (%)				<0.001
No	9,446 (76.5)	8,592 (75.9)	854 (82.9)	
Yes	2,906 (23.5)	2,730 (24.1)	176 (17.1)	
Smoking status, *n* (%)				<0.001
Never	6,443 (52.2)	6,039 (53.3)	404 (39.2)	
Former	3,134 (25.4)	2,929 (25.9)	205 (19.9)	
Current	2,775 (22.5)	2,354 (20.8)	421 (40.9)	
Alcohol intake, *n* (%)				0.278
No	3,419 (27.7)	3,119 (27.5)	300 (29.1)	
Yes	8,933 (72.3)	8,203 (72.5)	730 (70.9)	
Diabetes, *n* (%)				<0.001
No	10,986 (88.9)	10,122 (89.4)	864 (83.9)	
Yes	1,366 (11.1)	1,200 (10.6)	166 (16.1)	
Hypertension, *n* (%)				<0.001
No	8,828 (71.5)	8,182 (72.3)	646 (62.7)	
Yes	3,524 (28.5)	3,140 (27.7)	384 (37.3)	
Arthritis, *n* (%)				<0.001
No	8,982 (72.7)	8,395 (74.1)	587 (57.0)	
Yes	3,370 (27.3)	2,927 (25.9)	443 (43.0)	
Heart disease, *n* (%)				<0.001
No	11,367 (92.0)	10,486 (92.6)	881 (85.5)	
Yes	985 (8.0)	836 (7.4)	149 (14.5)	
Pulmonary disease, *n* (%)				<0.001
No	10,199 (82.6)	9,480 (83.7)	719 (69.8)	
Yes	2,153 (17.4)	1842 (16.3)	311 (30.2)	
Liver disease, *n* (%)				<0.001
No	11,945 (96.7)	10,982 (97.0)	963 (93.5)	
Yes	407 (3.3)	340 (3.0)	67 (6.5)	
Cancer, *n* (%)				0.074
No	11,234 (90.9)	10,313 (91.1)	921 (89.4)	
Yes	1,118 (9.1)	1,009 (8.9)	109 (10.6)	
Moisture intake, *n* (%)				0.002
Lowest quartile	3,088 (25.0)	2,783 (24.6)	305 (29.6)	
Middle lower quartile	3,088 (25.0)	2,861 (25.3)	227 (22.0)	
Middle upper quartile	3,088 (25.0)	2,847 (25.1)	241 (23.4)	
Highest quartile	3,088 (25.0)	2,831 (25.0)	257 (25.0)	
Total fat intake, *n* (%)				<0.001
Lowest quartile	3,088 (25.0)	2,769 (24.5)	319 (31.0)	
Middle lower quartile	3,088 (25.0)	2,851 (25.2)	237 (23.0)	
Middle upper quartile	3,088 (25.0)	2,840 (25.1)	248 (24.1)	
Highest quartile	3,088 (25.0)	2,862 (25.3)	226 (21.9)	
Protein intake, *n* (%)				<0.001
Lowest quartile	3,085 (25.0)	2,721 (24.0)	364 (35.3)	
Middle lower quartile	3,091 (25.0)	2,831 (25.0)	260 (25.2)	
Middle upper quartile	3,088 (25.0)	2,879 (25.4)	209 (20.3)	
Highest quartile	3,088 (25.0)	2,891 (25.5)	197 (19.1)	
Dietary fiber intake, *n* (%)				<0.001
Lowest quartile	3,071 (24.9)	2,697 (23.8)	374 (36.3)	
Middle lower quartile	3,083 (25.0)	2,854 (25.2)	229 (22.2)	
Middle upper quartile	3,102 (25.1)	2,865 (25.3)	237 (23.0)	
Highest quartile	3,096 (25.1)	2,906 (25.7)	190 (18.4)	
Carbohydrate intake, *n* (%)				0.007
Lowest quartile	3,088 (25.0)	2,786 (24.6)	302 (29.3)	
Middle lower quartile	3,088 (25.0)	2,854 (25.2)	234 (22.7)	
Middle upper quartile	3,088 (25.0)	2,848 (25.2)	240 (23.3)	
Highest quartile	3,088 (25.0)	2,834 (25.0)	254 (24.7)	
SSRI use, *n* (%)				<0.001
No	11,575 (93.7)	10,725 (94.7)	850 (82.5)	
Yes	777 (6.3)	597 (5.3)	180 (17.5)	
Constipation, *n* (%)				<0.001
No	11,922 (96.5)	10,982 (97.0)	940 (91.3)	
Yes	430 (3.5)	340 (3.0)	90 (8.7)	

PIR, poverty income ratio; BMI, body mass index; SSRI, selective serotonin reuptake inhibitor use.

### 3.2. Association between constipation and major depression

Based on univariate logistic regression analysis, race and ethnicity, sex, age, marital status, education level, PIR, BMI, smoking status, dietary intake, vigorous physical activity, SSRI use, and comorbidities (except cancer) were significantly related to major depression (all *p* < 0.05) (Table 3). In Table 4, the logistic regression results for the association between constipation and major depression are presented. The unadjusted model (Model I) showed an increased risk of major depression related to constipation (OR: 3.09, 95%CI: 2.43–3.94, *p* < 0.001). After controlling for sex and age (Model II), constipation and major depression were still associated (OR: 2.60, 95%CI: 2.03–3.32, *p* < 0.001). After adjusting for covariates in Model II and race/ethnicity, marital status, education level, PIR, BMI, vigorous physical activity, smoking status, SSRI use and alcohol consumption (Model III), constipation was still significantly positively associated with major depression (OR: 2.42, 95%CI: 1.86–3.14, *p* < 0.001). After controlling for covariates in Model III and diabetes, hypertension, arthritis, heart disease, pulmonary disease, liver disease, and cancer (Model IV), the results did not change significantly (OR: 2.29, 95%CI: 1.76–2.99, *p* < 0.001). Even after further adjusting for covariates in Model IV and dietary fiber, moisture, total fat, carbohydrate, and protein intake (Model V), constipation and major depression remained significantly associated (OR: 2.20, 95%CI: 1.68–2.87, *p* < 0.001).

**Table 3:** Univariate regression analysis.

Variable	OR_95CI	*p*_value
Age	0.993 (0.989–0.996)	<0.001
Sex: Female vs. Male	1.85 (1.62–2.11)	<0.001
Race/Ethnicity: ref. = White		
Black	1.25 (1.06–1.47)	0.009
Hispanic	1.27 (1.09–1.48)	0.002
Other	1.00 (0.71–1.43)	0.984
Marital Status: ref. = Married or living with partner		
Divorced, separated, or widowed	2.03 (1.75–2.35)	<0.001
Never married	1.57 (1.32–1.86)	<0.001
Education Level: > high school vs. ≤ high school	0.56 (0.49–0.64)	<0.001
Family PIR:≥ 2 vs. <2	0.32 (0.28–0.37)	<0.001
BMI: ref. = Under/normal weight (<25.0)		
Overweight (25.0–29.9)	0.89 (0.75–1.06)	0.203
Obese (>30.0)	1.43 (1.22–1.67)	<0.001
Vigorous physical activity: Yes vs. No	0.65 (0.55–0.77)	<0.001
Smoking status: ref. = Never		
Former	1.05 (0.88–1.24)	0.61
Current	2.67 (2.31–3.09)	<0.001
Alcohol intake: Yes vs. No	0.93 (0.80–1.06)	0.279
Diabetes: Yes vs. No	1.62 (1.36–1.93)	<0.001
Hypertension: Yes vs. No	1.55 (1.36–1.77)	<0.001
Arthritis: Yes vs. No	2.16 (1.90–2.47)	<0.001
Heart disease: Yes vs. No	2.12 (1.76–2.56)	<0.001
Pulmonary disease: Yes vs. No	2.23 (1.93–2.57)	<0.001
Liver disease: Yes vs. No	2.25 (1.72–2.94)	<0.001
Cancer: Yes vs. No	1.21 (0.98–1.49)	0.074
Moisture intake: ref. = Lowest quartile		
Middle lower quartile	0.72 (0.60–0.87)	<0.001
Middle upper quartile	0.77 (0.65–0.92)	0.004
Highest quartile	0.83 (0.70–0.99)	0.034
Total fat intake: ref. = Lowest quartile		
Middle lower quartile	0.72 (0.61–0.86)	<0.001
Middle upper quartile	0.76 (0.64–0.90)	0.002
Highest quartile	0.69 (0.57–0.82)	<0.001
Protein intake: ref. = Lowest quartile		
Middle lower quartile	0.69 (0.58–0.81)	<0.001
Middle upper quartile	0.54 (0.45–0.65)	<0.001
Highest quartile	0.51 (0.43–0.61)	<0.001
Dietary fiber intake: ref. = Lowest quartile		
Middle lower quartile	0.58 (0.49–0.69)	<0.001
Middle upper quartile	0.60 (0.50–0.71)	<0.001
Highest quartile	0.47 (0.39–0.57)	<0.001
Carbohydrate intake: ref. = Lowest quartile		
Middle lower quartile	0.76 (0.63–0.90)	0.002
Middle upper quartile	0.78 (0.65–0.93)	0.005
Highest quartile	0.83 (0.69–0.98)	0.033
SSRI use: Yes vs. No	3.80 (3.18–4.56)	<0.001
Constipation: Yes vs. No	3.09 (2.43–3.94)	<0.001

PIR, poverty income ratio; BMI, body mass index; SSRI, selective serotonin reuptake inhibitor use.

**Table 4:** Multivariate regression analysis of the association between constipation and major depression.

Variable	Normal OR (95%CI)	Constipated OR (95%CI)	*p*-value
Model I^a^	1 (Ref)	3.09 (2.43–3.94)	<0.001
Model II^b^	1 (Ref)	2.60 (2.03–3.32)	<0.001
Model III^c^	1 (Ref)	2.42 (1.86–3.14)	<0.001
Model IV^d^	1 (Ref)	2.29 (1.76–2.99)	<0.001
Model V^e^	1 (Ref)	2.20 (1.68–2.87)	<0.001

a Model I: no adjusted.

b Model II: adjusted for age + sex.

c Model III: Model II+ race/ethnicity + marital status + education level + poverty income ratio + body mass index + vigorous physical activity + smoking status + alcohol consumption + selective serotonin reuptake inhibitor use.

d Model IV: Model III + diabetes + hypertension + arthritis + heart disease + pulmonary disease + liver disease + cancer.

e Model V: Model IV + dietary fiber + moisture + total fat + carbohydrate + protein.

**Figure 2:**
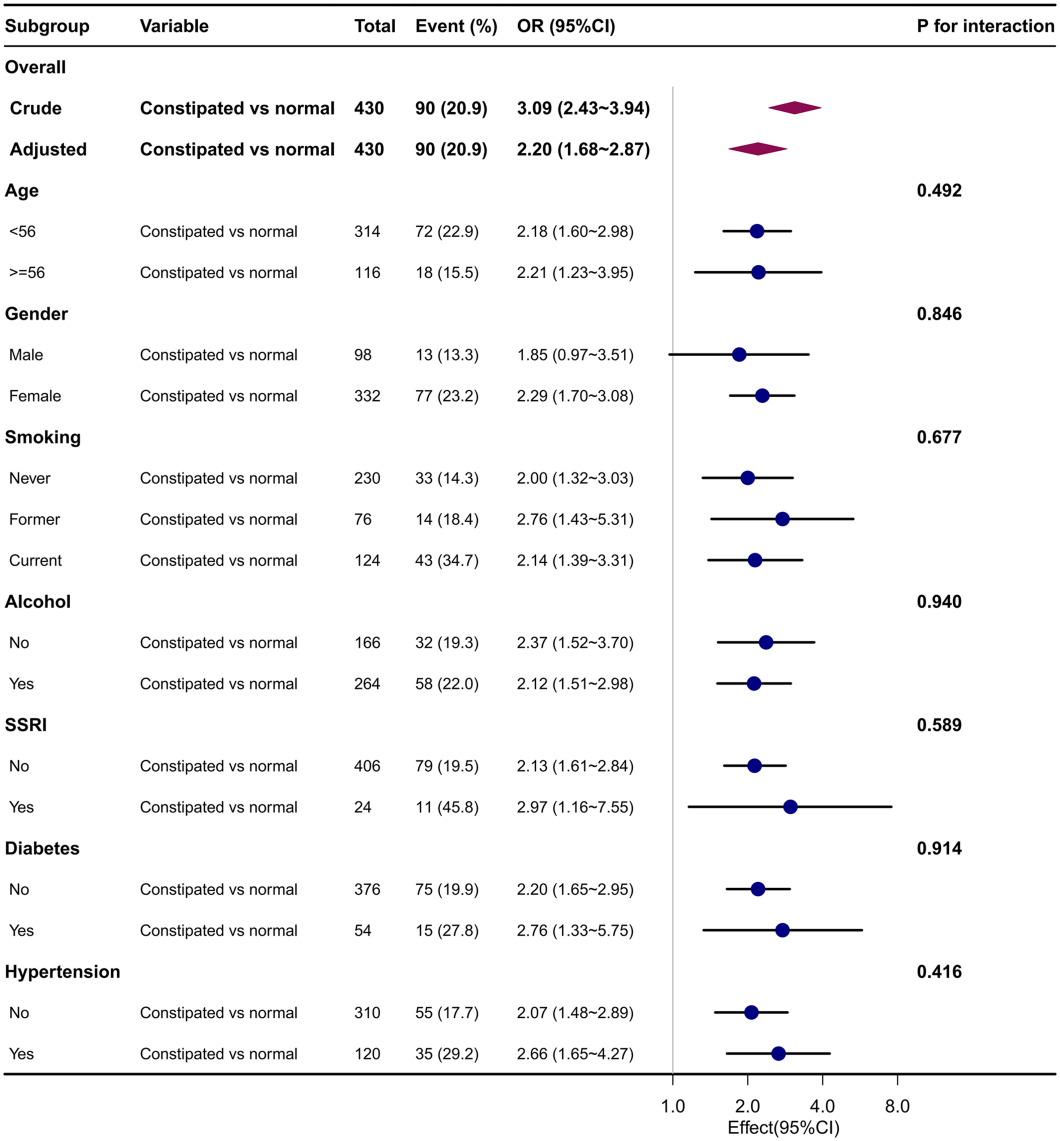
Association between constipation and major depression in different subgroups. Adjusted for age, sex, race/ethnicity, marital status, education level, vigorous physical activity, body mass index, family poverty income ratio, smoking status, alcohol intake, selective serotonin reuptake inhibitor use, diabetes, liver disease, heart disease, pulmonary disease, hypertension, arthritis, cancer, dietary fiber intake, moisture intake, total fat intake, carbohydrates intake, and protein intake.

A subgroup analysis of the data is presented in Figure 2 and Supplementary Figure 1. Subgroup analyses by age, sex, dietary intake, risk behaviors, SSRI use, and common complications showed no statistically significant interactions (*p* > 0.05). We found that the association between constipation and depression was relatively stable in every subgroup.

## 4. Discussion

Based on a nationally representative survey, we found that people with constipation had significantly higher rates of comorbid major depression than those without it. Subgroup analyses by age, sex, dietary intake, risk behaviors, and common complications showed no statistically significant interactions, and we found that the association between constipation and depression was stable in every subgroup.

One study, also based on the NHANES database (2009–2010 data were used), found that constipation was significantly associated with mild depression but not major depression (11). In that cited literature, constipation was defined by stool consistency based on the Bristol Stool Form Scale (BSFS). In our study, however, we found that major depression and constipation were also significantly associated. In addition, our sample size was larger because our data were employed from the NHANES 2005–2010. Finally, we defined constipation by having fewer than 3 stools per week, which was another validated method of defining constipation in the NHANES database. Different definitions of constipation, sample sizes and not exactly the same adjusted covariates may be the main reasons for the different results.

Among Iranian adults aged 18–55, a cross-sectional study evaluated the factors associated with chronic constipation and found depression to be significantly influential, with an OR and 95%CI of 1.69 (1.37–2.09) (10). And men were more likely to have this association than women (OR: 2.28, 95%CI: 1.50–3.63 vs. OR: 1.55, 95%CI: 1.21–1.99). Our findings were consistent with those described above in the population aged 55 years and younger. But our study further confirmed that constipation remained significantly associated with depression in individuals over 55 years of age. And we found that women were more likely to have this association than men (OR: 2.29, 95%CI: 1.70–3.08 vs. OR: 1.85, 95% CI: 0.97–3.51), which was contrary to the result of the above study. We believe that one of the reason for the inconsistency between our results and the results of the cited literature may be the difference in the study population. The study population in the cited literature is Iranian population in Asia, while our study population based on NHANES data is American population. Although no studies have shown that the strength of the association between constipation and depression differs between men and women in different populations, the possibility cannot be ruled out. In addition, different definitions of constipation may have contributed to the different results. In this cited literature, constipation was defined as chronic constipation according to Rome III criteria, and patients with IBS-constipation type (IBS-C) were excluded (10). In NHANES, however, our definition of constipation based on previous literature (12, 13) did not take into account abdominal pain, so patients with IBS cannot be identified and excluded (8). A meta-analysis of clinical studies on IBS has found that IBS-C type is more common in women (women, 40%; men, 21%; OR, 2.38; 95%CI, 1.45–3.92) (27), while depressive symptoms are also more common in IBS women [35.1% (95%CI: 23.0–47.3)] than in men [25.9% (95%CI: 11.9–39.9)] (28). Regardless of the reason for the different results, the significant association between constipation and depression was stable, and the differences in this association between sexes need to be further explored.

The association between constipation and depression may be related to the interaction between the central nervous system and the gastrointestinal tract. For example, corticotropin-releasing factor (CRF) is an important mediator of the relationship between emotional distress and changes in both upper and lower gastrointestinal (GI) motor function (29, 30). In functional GI disorders including constipation, dysfunction of the autonomic nervous system, which acts directly on CRF, may play a role in alteration in bowel habits and gastric emptying (31). Similarly, depression is associated with hyperactivity of CRF neuronal pathways (32) and CRF receptors have been suggested as a possible treatment target for both depression and GI disorders (33, 34). It is possible that consistent activation of the stress pathways mentioned above may lead to dysfunction in the brain-gut axis, making patients with constipation more likely to be complicated with depressive symptoms.

There are several strengths to the present study. Our first advantage was that we had a large, nationally representative sample of the American people by combining all the available cycles of continuous NHANES, allowing us to specifically study the association of constipation with major depression. Second, we adjusted for as many covariates as possible, such as income, health behaviors, dietary intake and comorbidities, which makes our results more robust. Finally, subgroup analyses were performed according to age, sex, dietary intake, risk behaviors, SSRI use, and common complications, which further validated the stability of our results.

However, a few limitations must also be noted in our study. First, as the study is cross-sectional, causal inferences regarding constipation and depression are not feasible, and reverse causality is also possible. Second, some covariables were left out of the analysis since no data was available for all volunteers (for example, the use of laxatives). Third, based on previous literature, we defined constipation by defecation frequency and could not determine whether participants met the Rome criteria for constipation. Last but not least, even after controlling for potential confounders, observational studies are susceptible to residual confounding.

In conclusion, this study showed that people with constipation had significantly higher rates of comorbid major depression than those without it. Constipation is significantly associated with major depression, suggesting that clinicians should pay close attention to the emotional and psychological status of patients. There is a need for depression screening and assessment in patients with long-term constipation, which can help find early or potential depressed patients and carry out timely and effective intervention.

## Data availability statement

The original contributions presented in the study are included in the article/Supplementary material, further inquiries can be directed to the corresponding authors.

## Ethics statement

The studies involving human participants were reviewed and approved by the National Center for Health Statistics Ethics Review Board of the Centers for Disease Control and Prevention. The patients/participants provided their written informed consent to participate in this study.

## Author contributions

PW: contributed to study planning, data analyses, and drafting of the manuscript. YW, XS, and XJ: contributed to study planning and manuscript development. All authors contributed to the article and approved the submitted version.

## Conflict of interest

The authors declare that the research was conducted in the absence of any commercial or financial relationships that could be construed as a potential conflict of interest.

## Publisher’s note

All claims expressed in this article are solely those of the authors and do not necessarily represent those of their affiliated organizations, or those of the publisher, the editors and the reviewers. Any product that may be evaluated in this article, or claim that may be made by its manufacturer, is not guaranteed or endorsed by the publisher.

## Supplementary material

SUPPLEMENTARY FIGURE 1 Association between constipation and major depression in dietary intake subgroups. Adjusted for age, sex, race/ethnicity, marital status, education level, vigorous physical activity, body mass index, family poverty income ratio, smoking status, alcohol intake, selective serotonin reuptake inhibitor use, diabetes, liver disease, heart disease, pulmonary disease, hypertension, arthritis, cancer, dietary fiber intake, moisture intake, total fat intake, carbohydrates intake, and protein intake.
