# Supplementary figures and images for: Association between constipation and major depression in adult Americans: evidence from NHANES 2005–2010
Source: Front Psychiatry. 2023 Aug 15;14:1152435. doi: 10.3389/fpsyt.2023.1152435 (PMC10465693; doi:10.3389/fpsyt.2023.1152435)

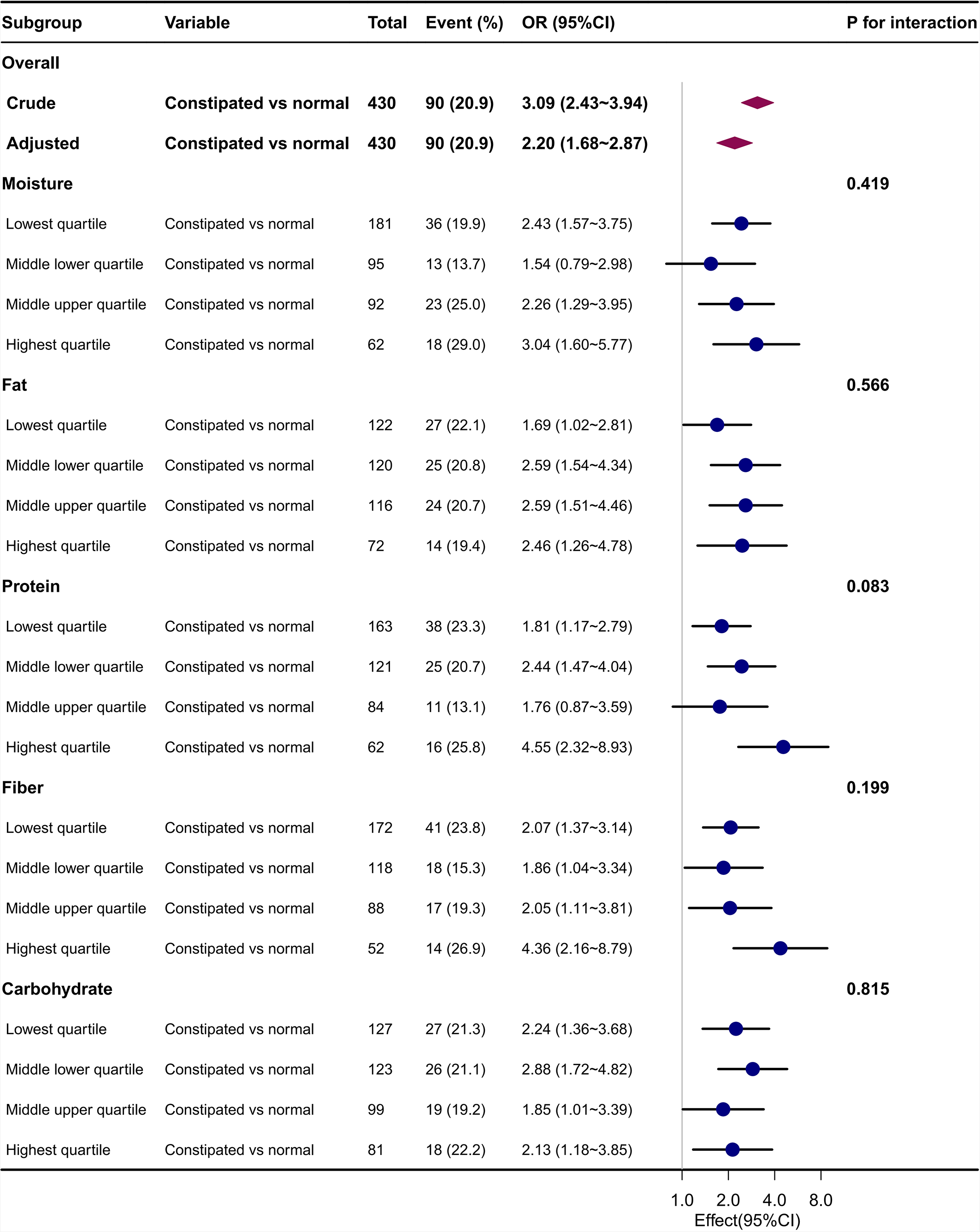

Supplement: SUPPLEMENTARY FIGURE 1 — Association between constipation and major depression in dietary intake subgroups. Adjusted for age, sex, race/ethnicity, marital status, education level, vigorous physical activity, body mass index, family poverty income ratio, smoking status, alcohol intake, selective serotonin reuptake inhibitor use, diabetes, liver disease, heart disease, pulmonary disease, hypertension, arthritis, cancer, dietary fiber intake, moisture intake, total fat intake, carbohydrates intake, and protein intake. [file image_1.tif]
